# Surgical Approach to the Cavernous Sinus for a Trigeminal Schwannoma Resection: Technical Note and Case Report

**DOI:** 10.1155/2016/6458189

**Published:** 2016-10-10

**Authors:** Riccardo Caruso, Alessandro Pesce, Venceslao Wierzbicki, Luigi Marrocco, Emanuele Piccione

**Affiliations:** ^1^Neurosurgery Department, Celio Military Hospital, Rome, Italy; ^2^NESMOS Department, Sapienza University of Rome, Rome, Italy; ^3^Neurosurgery Department, Azienda Ospedaliera Sant'Andrea, Rome, Italy

## Abstract

We report a rare case of schwannoma of the lateral wall of the cavernous sinus, an exceedingly rare lesion affecting this anatomical district, and discuss salient aspects of the surgical approach to the cavernous sinus, which are traditionally considered technically challenging due to the high risk of postoperative morbidity and mortality related to the presence of the cranial nerves and internal carotid artery.

## 1. Introduction

Schwannomas of the cavernous sinus are very rare and usually originate from the trigeminal nerve, though cases originating from the oculomotor nerves and from the carotid plexus have also been described in the literature [1–10]. In this article, we report the case of a schwannoma of the lateral wall of the cavernous sinus that was surgically treated.

## 2. Case Presentation

In July 2013, a 39-year-old man came to our attention: for three months he had been complaining of diplopia when looking sideways to the right. In the last two weeks before his examination he had also been suffering from frequent episodes of right-sided hemifacial paraesthesias. Upon neurological examination, it was revealed that there was a deficit of the 6th cranial nerve on the right side, which arose only when the patient looked sideways, to the far right.

**Figure 1:**
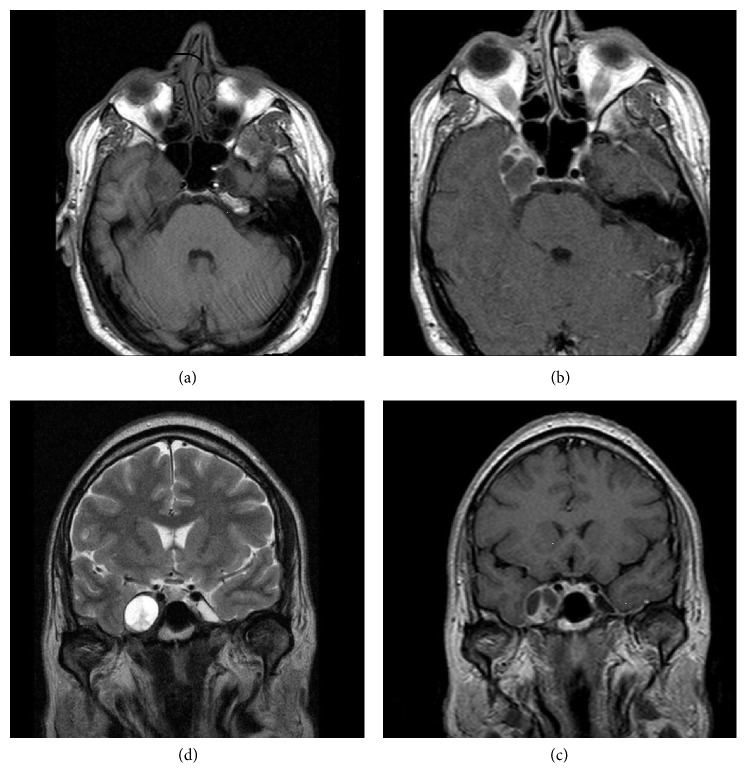
One can see clockwise the MR images of the tumour on T1-weighted, on T1 with contrast, and on T2-weighted images.

The patient had undergone a cerebral MRI scan (Figure 1) that showed a 3 cm maximal diameter isointense lesion centred in the region of the right cavernous sinus on T1-weighted images. In T2-weighted images, the lesion was hyperintense compared with the surrounding brain. The lesion presented inhomogeneous enhancement after contrast injection.

**Figure 2:**
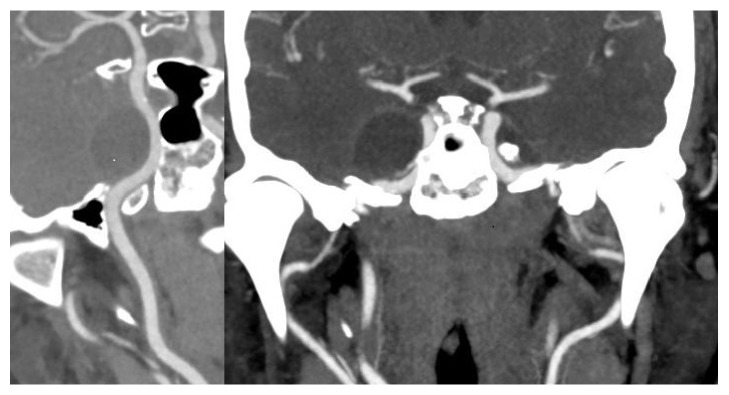
The CT angiography shows that the tumour is adjacent to the wall of the right internal carotid artery.

**Figure 3:**
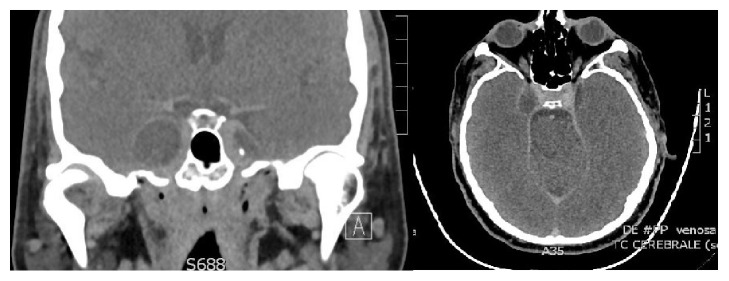
The CT venous phase shows that the cavernous sinus is compressed and medialized by the tumour rather than invaded by it.

**Figure 4:**
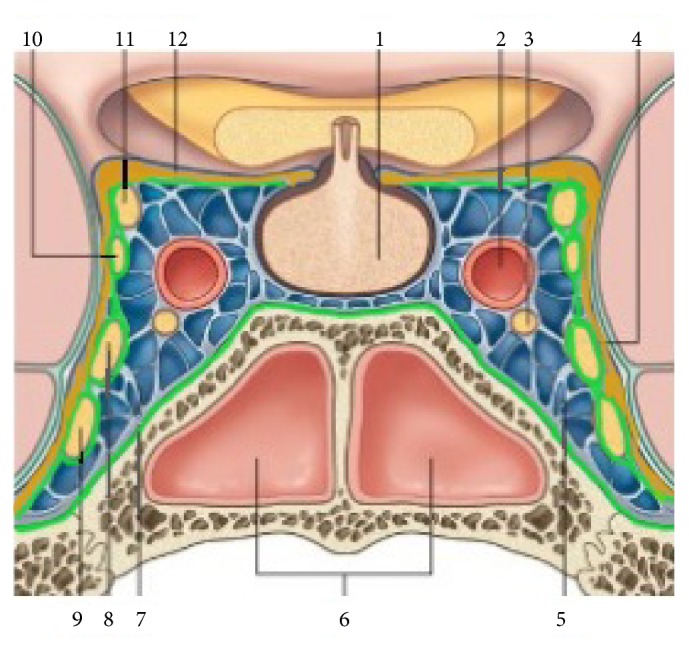
Anatomical diagram of the cavernous sinus: 1: hypophysis, 2: internal carotid artery, 3: 6th CN, 4: meningeal (outer) layer, 5: cavernous sinus, 6: sphenoid sinus, 7: endosteal (inner) layer, 8: V_1_, 9: V_2_, 10: 4th CN, 11: 3rd CN, and 12: the outer layer at the diaphragma sellae. The endosteal layer is marked in green, while the meningeal layer is marked in yellow.

While in hospital, a CT angiography was performed in order to document the relationship of the tumour with the ICA and with the cavernous sinus. The mass appeared adjacent to the wall of the carotid artery (Figure 2); the CT venous phase (Figure 3) showed that the cavernous sinus was compressed and medialized by the tumour rather than invaded by it. On the basis of the images and of the clinical history of the patient it was theorized that the tumour might have developed in the virtual space existing between the dura and the inner membrane of the lateral wall of the cavernous sinus (Figure 4).

**Figure 5:**
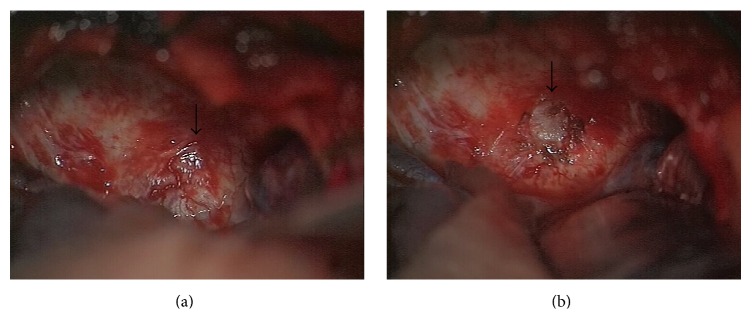
In (a), the arrow indicates the bulging of the lateral wall of the cavernous sinus caused by the tumour; in (b), the arrow points to the appearance of the tumour after the incision of the dura.

The patient underwent microneurosurgery using the pterional approach; we opened the dura, moved with a spatula the medial part of the temporal pole, and exposed the lateral wall of the cavernous sinus, which was severely bulging especially in the lower part. The dura of the wall of the cavernous sinus was incised where it protruded the most, thus exposing a well-capsulized grey-yellowish tumour (Figure 5). With an ultrasonic aspirator the tumour was partially emptied, thus also draining two small intratumoural cysts full of yellow fluid; then, the cleavage of the capsule was performed. The cleavage was quite easy both in the lateral and in the inferior parts where the capsule was adjacent to the dura, but it became more difficult in the middle as there was a very thin, pulsing membrane that in some areas adhered to the tumour.

Once the dissection was completed, the tumour, already partially emptied, was removed en bloc. After the removal, there was a slight leakage of arterial blood from the inner membrane; however, haemostasis was easily achieved by applying a gelatin matrix hemostatic sealant into the cavity.

**Figure 6:**
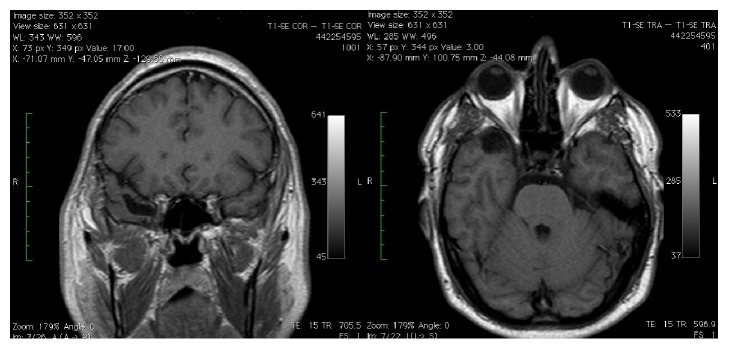
An MRI scan performed 5 months after surgery shows complete removal of the tumour.

The postoperative course was normal and, in the first few hours after surgery, the 6th CN deficit and the paresthesias disappeared. The histological examination showed that the tumour was a schwannoma. An MRI scan (Figure 6) performed 5 months after surgery showed complete removal of the tumour.

Before writing this paper, the patient was consulted and gave informed written explicit consent to this report.

## 3. Discussion

Apart from its rarity, we want to stress two features that make our case interesting: a clinical feature and the surgical technique used.

**Figure 7:**
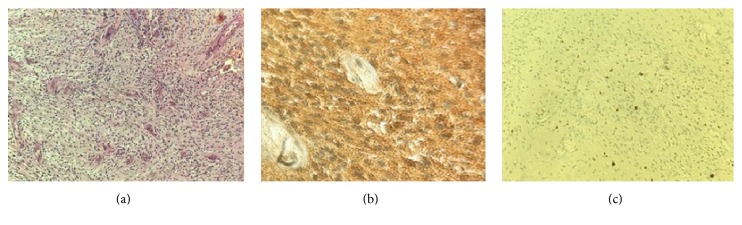
(a) Hematoxylin and Eosin, 20x: the classical histopathology of a schwannoma, hypercellular matrix composed of spindle cells with elongated nuclei, (b) with strong positive S100 immunostaining, and (c) rare mitotic figures outlined with Ki-67 immunostaining.

We believe that the tumour we operated on was a trigeminal schwannoma (Figure 7), even though the first symptom was a deficit of the 6th CN. The scans, in fact, show that the tumour was growing in the space between the dura mater and the inner membrane of the cavernous sinus lateral wall where the branches of the trigeminal nerve run, while the 6th CN runs inside the venous sinus [11–13]. In the literature there are other cases of neurinoma of the 5th CN with a clinical onset of palsy in the 6th CN [14].

From the anatomical viewpoint, the lateral wall of the cavernous sinus, which is more properly named* paraclinoid area*, is composed of a fold of the two layers of the dura mater: the endosteal and meningeal layers. In this region, the meningeal layer represents the outer layer of the cavernous sinus, while the endosteal one is the inner layer and thus envelops the intracavernous course of the 3rd, 4th, and 5th CN [15].

The exact localization of the tumour in what is usually a nominal area between the dura mater of the outer layer and the inner membrane made us carry out the surgery of the schwannoma not by entering the cavernous sinus through the Parkinson triangle [16, 17] but by attacking it directly by opening the dura where it bulged out the most. This surgical choice, together with the caution in dissecting the tumoural capsule from the inner membrane, allowed us to remove the tumour while remaining outside of the venous sinus and therefore avoiding the copious bleeding that makes performing surgery on this area particularly difficult. This way we also managed to preserve the various cranial nerves without having to expose them and isolate them one by one. The ICA trunk was not exposed but simply perceived through the pulsation of the inner membrane. We chose an intradural approach, but we could have achieved the same surgical results with an epidural approach [18–20].

In conclusion, our choice of surgical technique was successful and the removal of the tumour was fairly uncomplicated.

## Abbreviations

ICA: Internal carotid artery
MRI: Magnetic Resonance Imaging
CN: Cranial nerve.
